# Identification and correction of spatial bias are essential for obtaining quality data in high-throughput screening technologies

**DOI:** 10.1038/s41598-017-11940-4

**Published:** 2017-09-20

**Authors:** Bogdan Mazoure, Robert Nadon, Vladimir Makarenkov

**Affiliations:** 1 Department of Computer Science, McGill University, Montreal, Canada; 2 Department of Human Genetics, McGill University, Montreal, Canada; 3 McGill University and Genome Quebec Innovation Centre, Montreal, Canada; 4 Department of Computer Science, Université du Québec à Montréal, Montreal, Canada

## Abstract

Spatial bias continues to be a major challenge in high-throughput screening technologies. Its successful detection and elimination are critical for identifying the most promising drug candidates. Here, we examine experimental small molecule assays from the popular ChemBank database and show that screening data are widely affected by both assay-specific and plate-specific spatial biases. Importantly, the bias affecting screening data can fit an additive or multiplicative model. We show that the use of appropriate statistical methods is essential for improving the quality of experimental screening data. The presented methodology can be recommended for the analysis of current and next-generation screening data.

## Introduction

High-throughput screening (HTS) is a widely used drug discovery technique which allows researchers to rapidly conduct millions of chemical, genetic or pharmacological experiments^1,2^. HTS technology relies on robotic handling systems, liquid handling systems, data mining tools and control software in order to assess the biological or biochemical activity of a large number of chemical compounds. Using HTS, researchers can discover new active compounds, antibodies or genes modulating a certain biomolecular pathway^3–6^. Growing needs of the modern pharmaceutical industry have motivated the recent advances in data throughput and data quality of high-throughput screening campaigns^3^. Processing hundreds of thousands compounds a day has become routine in screening laboratories worldwide. A typical HTS assay consists of a library of chemical compounds which are screened against the selected biological target in order to discover potential drug candidates, called hits^2^. An HTS library, organized according to biological activity (e.g. small molecules, siRNA or shRNA) or target specificity (e.g. enzymes such as kinases, proteases or phosphatases) is arrayed into micro-well plates enabling screening in a miniaturized form – in 96, 384, 1536 or 3456-well plates.

Unfortunately, experimental high-throughput screens are usually affected by spatial bias (i.e., systematic error) which negatively impacts the hit selection process^7–10^. Various sources of bias include reagent evaporation, cell decay, errors in liquid handling, pipette malfunctioning, variation in incubation time, time drift in measuring different wells or different plates, and reader effects^8,10,11^. Spatial bias is usually evident as row or column effects, especially on plate edges^7,9,12^. It produces over or under-estimation of true signals in specific rows or columns within the same plate and/or specific well locations across plates^8,9^. The presence of spatial bias in high-throughput screening assays can lead to an increase in the false positive and false negative rates during the hit identification process (see for example: a colorimetric immunoassay analyzed by Brideau *et al*.^7^; McMaster Data Mining and Docking Competition assay described in Elowe *et al*.^13^ and analyzed in Dragiev *et al*.^8^; RNAi HIV assay analyzed by Carralot *et al*.^14^ or LINCS gene expression assay analyzed by Lachmann *et al*.^10^). If they are not corrected using appropriate bias minimization methods, biased measurements can be falsely identified as hits, leading to an increase in both the length and the cost of the drug discovery process^2,10^.

ChemBank is a public small-molecule screens database which allows life scientists from various institutions to make their experimental screening data accessible to the research community^15^. The ChemBank project hosts 4,767 assays (as of November 30^th^, 2016) that cover a wide range of molecules, species and screening technologies. Small molecules are crucial components of a growing drug discovery toolbox used for studying cellular processes and developing effective therapies^16^. The three main screening technologies represented in ChemBank are high-throughput screening^17^ (HTS), high-content screening^18^ (HCS) and small-molecule microarrays^16^ (SMM). High content screening encompasses a set of analytical methods based on automated microscopy, multi-parameter image processing and visualization tools to identify substances such as small molecules, peptides or RNAi that alter the phenotype of a cell in a desired manner^18^. Thus, HCS can be viewed as a type of phenotypic screen conducted in cells. This technology normally uses fluorescence imaging of samples in a high-throughput format to extract quantitative data, such as spatial distribution of targets, or individual cell and organelle morphology, from cell populations. Microarray technique for screening small molecules is based on the use of machine-printed glass slides with an array of wells^16^. SMM is known to be an effective tool for discovering protein-small molecule interactions.

In this paper we analyze various data sets from ChemBank and show that most of them are affected by both assay-specific (when a certain bias pattern appears within all the plates of a given assay) and plate-specific (when a certain bias pattern appears within a given plate only) spatial biases. Furthermore, we provide evidence that spatial bias can be either additive or multiplicative^19^, depending in part on the screening technology. We show that both plate and assay-specific biases can be effectively identified and corrected. The correction of both of these biases is critical for the selection of quality hits^20^ in high-throughput screening campaigns.

## Results

### Simulation study

To assess the efficiency of our method, including both assay-specific bias correction by using robust Z-scores and plate-specific bias correction by using the additive and multiplicative PMP algorithms (see the Methods section), we compared its performance with those of the B-score^7^ and Well Correction^11,21^ methods, considering synthetic data with known hits and bias rates. The B-score method is the most known plate-specific correction method used in HTS, while Well Correction is an effective assay-specific correction technique removing systematic error from biased well locations. In our simulations, we followed the data simulation protocol applied by Dragiev *et al*.^8^ for testing methods minimizing additive spatial bias in HTS. First, 100 HTS assays, each including 50 plates of size (16 × 24), were generated (i.e., this is the most widely-used plate format in Chembank^15^) for each parameter combination described below.

Inactive compound measurements were sampled from the standard normal distribution. Hit (i.e., active compound) locations were selected independently for each plate using rejection sampling with the following hit percentages: 0.5%, 1%, 2%, 3%, 4% and 5%. Hit measurements were generated from the normal distribution with the following parameters ~*N*(*μ* − 6 *SD*, *SD*), where *μ* and *SD* were respectively the mean and the standard deviation of the inactive compound measurements (i.e., *μ* = 0 and *SD* = 1).

Well locations affected by assay-specific spatial bias were selected randomly with probability *p*_*a*_ = 0.29, estimated from the data taken from the ChemBank repository (see Tables 1 and 2 in Supplementary Information). Assay-specific bias was sampled from a normal distribution with parameters ~*N*(0, *C*), where the parameter *C* was taking the values: 0, 0.6 *SD*, 1.2 *SD*, 1.8 *SD*, 2.4 *SD*, and 3 *SD*, and added to the selected well locations. Plate-specific bias was then generated and added independently to rows and columns of each plate of a given assay. A maximum of 8 rows and 12 columns of each plate were allowed to be affected by spatial bias. For each plate of a given assay, the number of biased rows was sampled from a ~*Geometric*(*p* = 0.622) distribution and the number of biased columns was sampled from a ~*Geometric*(*p* = 0.430) distribution. These parameters were obtained using the maximum likelihood approach applied to the distributions of the number of biased rows and columns in the 2441 non-empty plates of the 175 ChemBank assays analyzed in this study (see Supplementary Figure 1 in Supplementary Information).

The plate-specific bias model for each plate was selected from: no bias, additive bias model and multiplicative bias model with the following respective sampling probabilities: 0.274, 0.418 and 0.308. These probabilities were also estimated from the considered ChemBank data (here, the no bias model included the probabilities of both undetermined and no bias models discussed in the next sections). The additive plate-specific bias was generated according to the normal distribution with parameters ~*N*(0, *C*) and the multiplicative plate-specific bias according to the normal distribution with parameters ~*N*(1, *C*), where the parameter *C* was taking the following values: 0, 0.6 *SD*, 1.2 *SD*, 1.8 *SD*, 2.4 *SD* and 3 *SD*. Plate measurements affected by additive spatial bias were generated using Equation (3) and plate measurements affected by multiplicative spatial bias were generated using Equation (4). A small Gaussian noise (i.e., random noise *ε*_*ijp*_ in Equations 3 and 4) was generated from the normal distribution with parameters ~*N*(0, 0.1 *SD*) and added to both active and inactive compound measurements of each plate.

Four bias correction methods were compared in our simulations: (1) No Correction, (2) B-score^7^, (3) Well-Correction^11^ and (4) our new method correcting both plate and assay-specific biases (i.e., additive or multiplicative PMP followed by robust Z-scores) as described in the Methods section. Moreover, both the Mann-Whitney *U* test and the Kolmogorov-Smirnov two-sample test, included in our method, were independently executed using the two following significance thresholds: *α* = 0.01 and *α* = 0.05 (this threshold was always the same for both tests).

**Figure 1:**
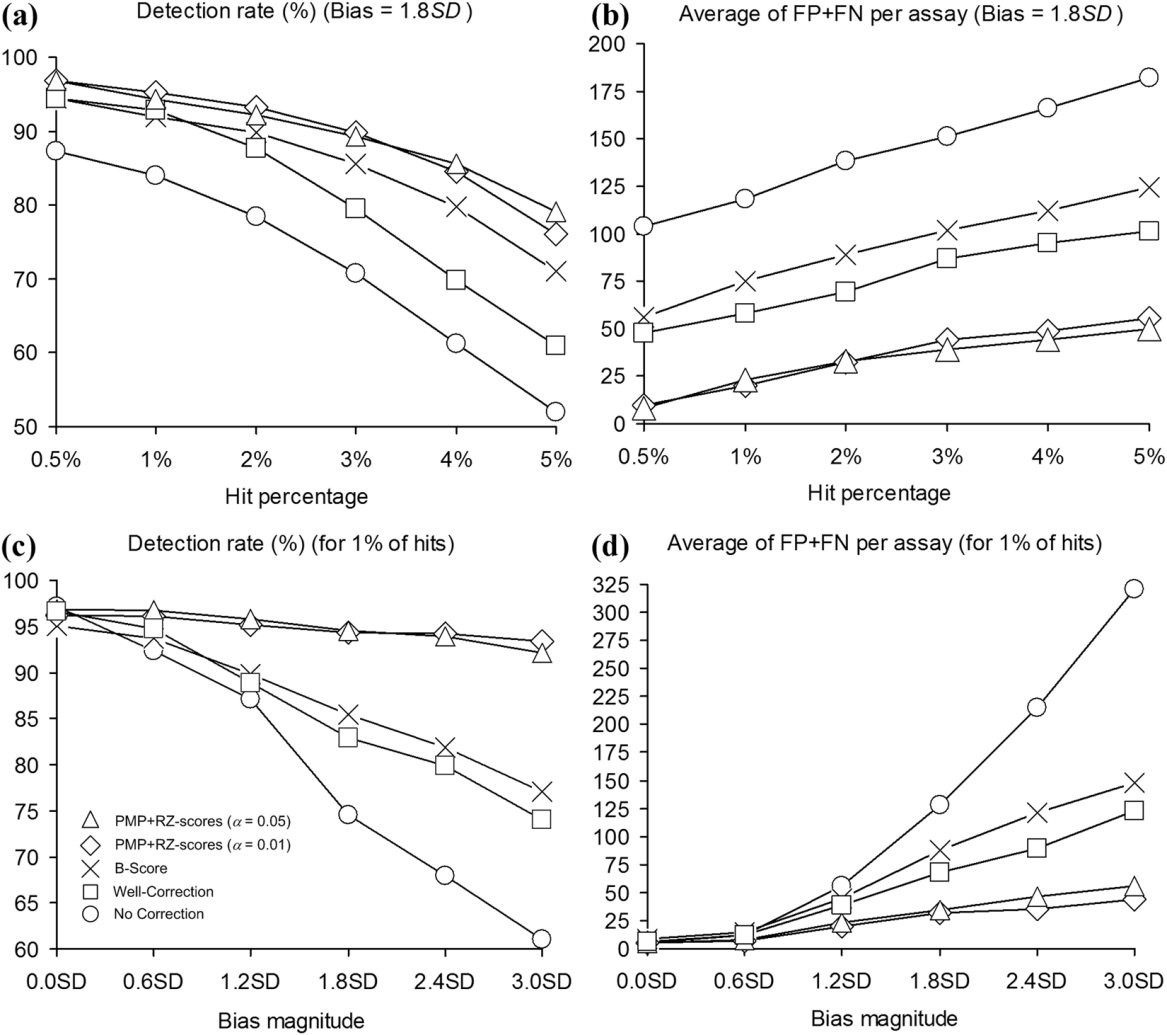
Average true positive rate (Panels (a) and (c)) and average total number of false positive and false negative hits per assay (Panels (b) and (d)) obtained by No Correction, Well Correction, B-score, and PMP followed by robust Z-scores for the significance levels *α* = 0.01 and 0.05. Panels (a) and (b) show the results obtained for datasets with a fixed bias magnitude (*SD* = 1.8). Panels (c) and (d) show the results for datasets with a fixed hit percentage of 1%.

After the data correction, hits were selected according to the *μ*_*p*_ − 3*σ*_*p*_ threshold, where *μ*_*p*_ and *σ*_*p*_ were the mean and the standard deviation of the measurements in plate *p*, respectively. We assessed the methods’ performances by comparing their hit detection rates (i.e., true positive rates) as well as by counting the total of false positives and false negatives they provided^8^. Two simulations were carried out for both selected values of *α*. In the first simulation, the bias magnitude, *SD*, was fixed to 1.8, while the hit percentage ranged from 0% to 5%. In the second simulation, the hit percentage was fixed to 1%, while the noise magnitude ranged from 0 to 3 *SD*. Fig. 1 shows the average true positive rate and the average total count of false positive and false negative hits per assay obtained for artificially generated HTS screens composed of 384-well plates, yielded by the competing methods (our method was carried out twice, using the significance levels *α* = 0.01 and *α* = 0.05, respectively).

The obtained results suggest that the additive and multiplicative PMP algorithms applied together with robust Z-scores yield the highest hit detection rate and the lowest false positive and false negative total hit count across all examined methods. When the hit percentage varies from 0.5% to 5%, and the bias magnitude is constant at 1.8 *SD* (Fig. 1a), the true positive rate for all methods decreases with the increase in the hit percentage. A similar trend in the true positive rate can be observed when the bias magnitude increases from 0.0 *SD* to 3.0 *SD* and the hit percentage remains fixed at 1% (Fig. 1c). It is worth noting that the PMP algorithm, followed by the robust Z-score normalization, has a very similar behaviour for both tested significance levels, *α* = 0.01 and *α* = 0.05 used in the Mann-Whitney *U* test and the Kolmogorov-Smirnov test. A slightly better hit recovery was obtained for the significance level *α* = 0.01 for lower hit percentages, and for the significance level *α* = 0.05 for higher hit percentages (Figs. 1a and 1b). When the hit percentage ranges from 0.5% to 5% and the bias magnitude stays constant at 1.8 *SD* (Fig. 1b), the average total count of false positives and false negatives increases for all methods. The PMP algorithm followed by assay-wise normalization by robust Z-scores provides the lowest total counts in all cases. When the bias magnitude varies from 0.0 *SD* to 3.0 *SD* and the hit percentage stays constant at 1%, our method still outperforms the three other competing approaches. The traditional additive B-score method^7^ usually copes well with the recovery of true positive hits (Fig. 1a and c), but provides a large number of false positives and thus gets outperformed by Well Correction^11^ in terms of the total number of false positive and false negative hits (Fig. 1b and d). In all cases, except the case of unbiased data (i.e., Bias = 0.0 *SD*) and the B-score method (Fig. 1c), the examined bias correction methods outperform the No Correction approach.

### Quantifying assay-specific bias in screening technologies

**Figure 2:**
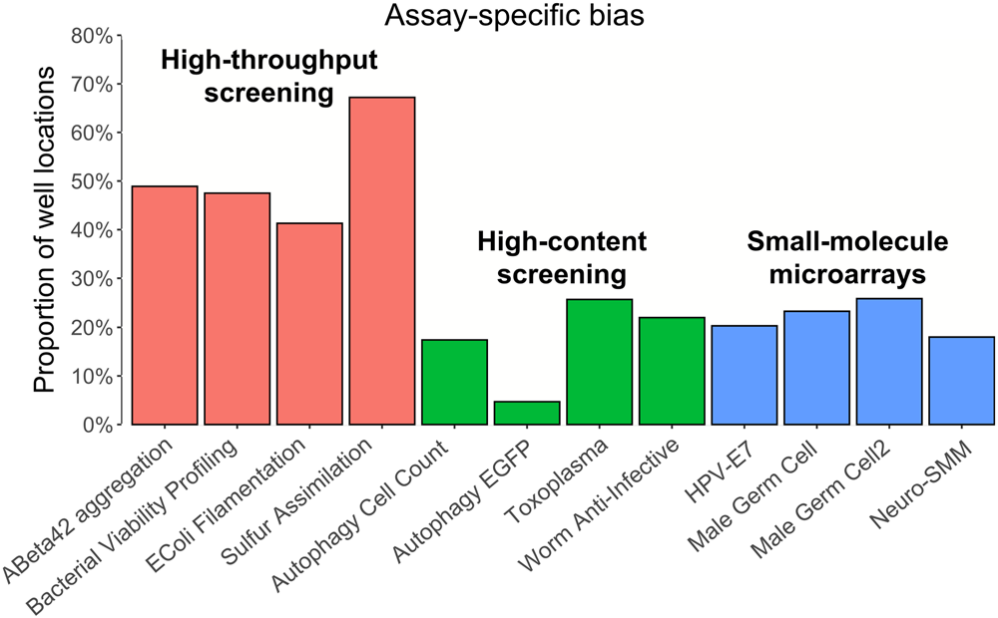
Assay-specific bias detected in 12 experimental assays from the ChemBank database. Here, 4 high-throughput screening assays, 4 high-content screening assays and 4 small-molecule microarrays were examined. The ChemBank IDs of these assays are indicated between parentheses: ABeta42 aggregation inhibitors (1103.0016), Bacterial viability profiling (1064.0002), *E. coli* filamentation (1038.0004), *M. tuberculosis* sulfur assimilation (130.0018), Autophagy cell count (1050.0009), Autophagy EGFP (1050.0111), Toxoplasma invasion imaging screening (141.0027), *C. elegans* assay for anti-infective reagents (1109.0003), HPV-E7 SMM (1049.0001), Male germ cell targets SMM (1154.0015), Male germ cell targets SMM (1154.0009) and NeuroSMM screen on torsin A (1069.0001); for more details see Supplementary Table 1.

To assess the extent of assay-specific bias in the HTS, HCS and SMM technologies, we examined 12 experimental assays from the ChemBank assays repository (i.e., 4 assays per technology were considered; Fig. 2). First, the data of the selected screens were normalized on a plate-by-plate basis using robust *Z*-scores. Second, the nonparametric Mann-Whitney *U* test was carried out independently for each well location (i.e., a vector of measurements taken across all plates of a given assay which corresponds to a fixed well position (*i,j*), where *i* is the row number and *j* is the column number), comparing its sum of ranks to the sum of ranks of the rest of the assay measurements. The significance level *α* = 0.01 was used in the Mann-Whitney *U* test (see the Methods section). Because the Mann-Whitney *U* test does not make any assumption about the underlying data distribution, its use has been recommended for bias detection purposes in screening technologies^22^. If the presence of spatial bias in a particular well location has been supported by the Mann-Whitney *U* test, then the traditional *Z*-score normalization should be carried out across its measurements. Importantly, the traditional *Z*-score normalization carried out across the measurements of a given well location can remove successfully both additive and multiplicative types of spatial bias. The detailed explanation of this property is presented in the Methods section.

Figure 2 shows that although all three small-molecule screening technologies are prone to assay-specific bias, it is much more substantial in HTS assays. According to the Mann-Whitney *U* test, 51.2% of well locations of HTS assays were affected by spatial assay-specific bias, whereas this bias affected only 17.4% and 21.8% of well locations in HCS and SMM screens, respectively.

### Quantifying plate-specific bias in screening technologies

**Figure 3:**
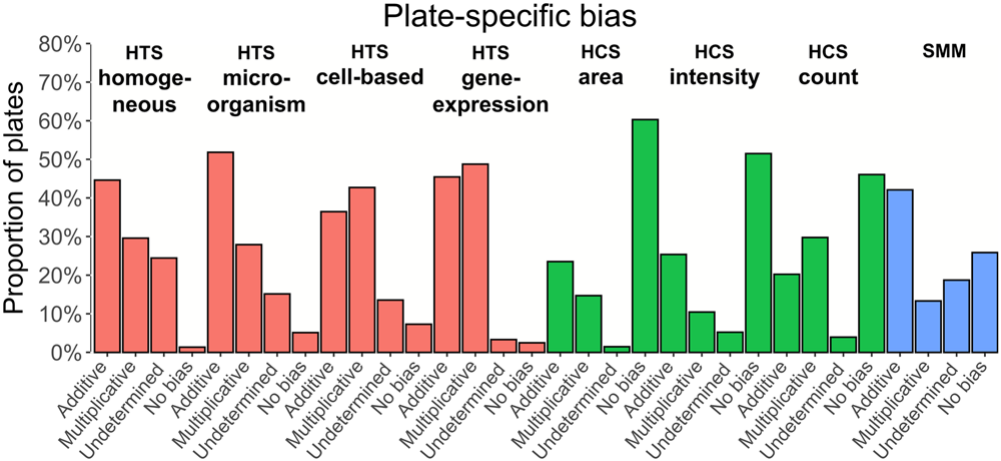
Plate-specific bias detected across data of 3 screening technologies and 8 screening categories available in ChemBank - per plate representation; 175 assays were analyzed in total (see Supplementary Table 2 for the complete list of the assays considered); all control wells were ignored.

**Figure 4:**
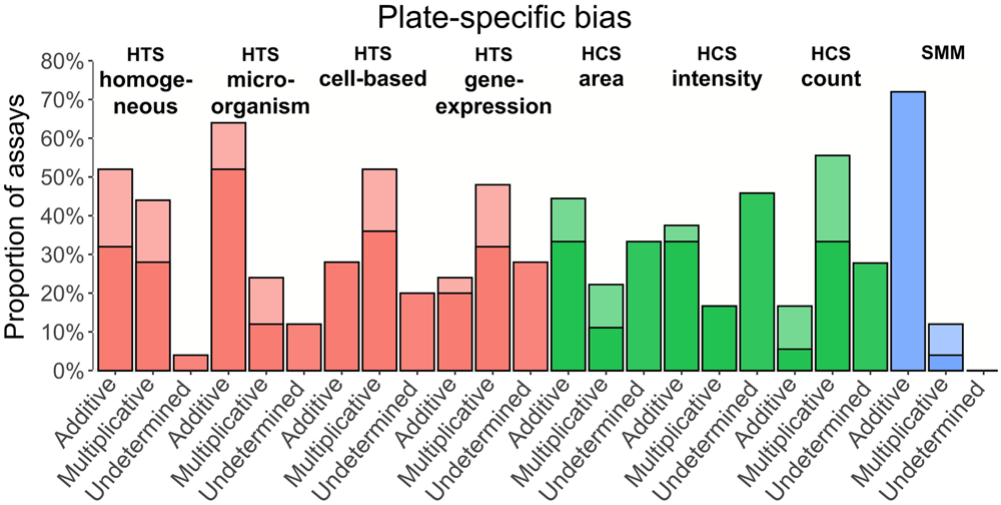
Plate-specific bias detected across data of 3 screening technologies and 8 screening categories available in ChemBank - per assay representation; 175 assays were analyzed in total (see Supplementary Table 2 for the complete list of the assays considered); all control wells were ignored. Assays, in which the number of plates containing additive bias was bigger than the number of plates containing multiplicative bias, are reported in the first column of each screening category. Assays with a bigger number of plates affected by multiplicative bias are reported in the second column of each screening category. Assays, in which the number of plates containing additive bias was equal to the number of plates containing the multiplicative bias as well as assays without any biased plate are reported as “Undetermined”. Darker portions of bars show the proportion of assays that have a dominant, additive or multiplicative, trend. Lighter portions of bars show the proportion of assays in which the indicated model of bias was present more frequently, but without a clear-cut dominance.

Differences among the bias distribution were also observed at the plate level (Figs 3 and 4). Plate-specific spatial bias is evident as systematic over or under-estimation of the measurements in specific rows and columns within specific plates. The Mann-Whitney *U* test (with *α* = 0.01) was performed independently on all plates of all assays considered in this study to identify the rows and columns affected by spatial bias (i.e., comparing the row and column measurements to unbiased data of the same plate). If systematic error was detected in one of the rows or columns of the plate, then additive and multiplicative Partial Mean Polish (PMP) bias algorithms^8^ were applied independently to correct the plate measurements. The measurements corrected by the additive and multiplicative PMP algorithms were then compared to unbiased plate measurements to select the best-fit correction model for the data at hand. The main advantages of the PMP algorithms are that they modify the values of the biased measurements only and keep the raw and corrected data on the same scale. The Kolmogorov-Smirnov test was performed on the plate surfaces corrected by the additive and multiplicative PMP algorithms and the model providing the smallest significant *p*-value was selected as the most appropriate (see the Methods section).

Figure 3 shows the *proportion of plates* per screening category affected by additive bias, multiplicative bias, an undetermined type of bias, or when no bias has been detected. An undetermined type of bias has been identified when spatial bias was detected on the plate by the Mann-Whitney *U* test and both *p-*values, corresponding to the additive and multiplicative models, were significant. The presented results suggest that the type of plate-specific bias largely depends on the screening category. For instance, additive bias is dominant in the following screening categories: HTS homogeneous (44.6%), HTS microorganism (51.8%) and SMM (42.1%) assays. The multiplicative bias is dominant in: HTS cell-based (36.5%) and HTS gene expression (45.5%) assays. However, all three HCS categories (area, intensity and cell count) were mostly bias free (Fig. 3).

Figure 4 presents the distribution of additive and multiplicative biases per assay, and per screening category. The *proportion of assays* affected either by additive bias, or by multiplicative bias, or by undetermined type of bias was represented for each screening category. Here, we also determined the proportions of assays in which the detected bias trend was dominant. The additive trend was considered dominant if there were twice or more biased plates better fitting the additive than the multiplicative bias model (and vice-versa for the multiplicative trend). The results presented in Fig. 4 suggest that in most cases one type of spatial bias was dominant in the ChemBank screens, the only exception here being the assays of the HCS count category affected by additive bias.

### Identifying spatial bias in the McMaster Data Mining and Docking Competition HTS Test assay

To illustrate the application of spatial bias correction methods, we carried out the assessment and correction of both assay-specific and plate-specific biases present in the McMaster Data Mining and Docking Competition HTS Test assay examined in several studies^8,11,13^. This assay was obtained during a screen of 50,000 diverse small molecules against a single target, dihydrofolate reductase enzyme of *E. coli*^19^. The McMaster Test assay consisted of a series of 625 plates of size (8 × 12). Each of the assay plates was screened twice. The first and the last columns of each plate contained controls that were used to normalize raw measurements. The remaining 80 wells contained different compounds intended to inhibit the dihydrofolate reductase of *E. coli*.

**Figure 5:**
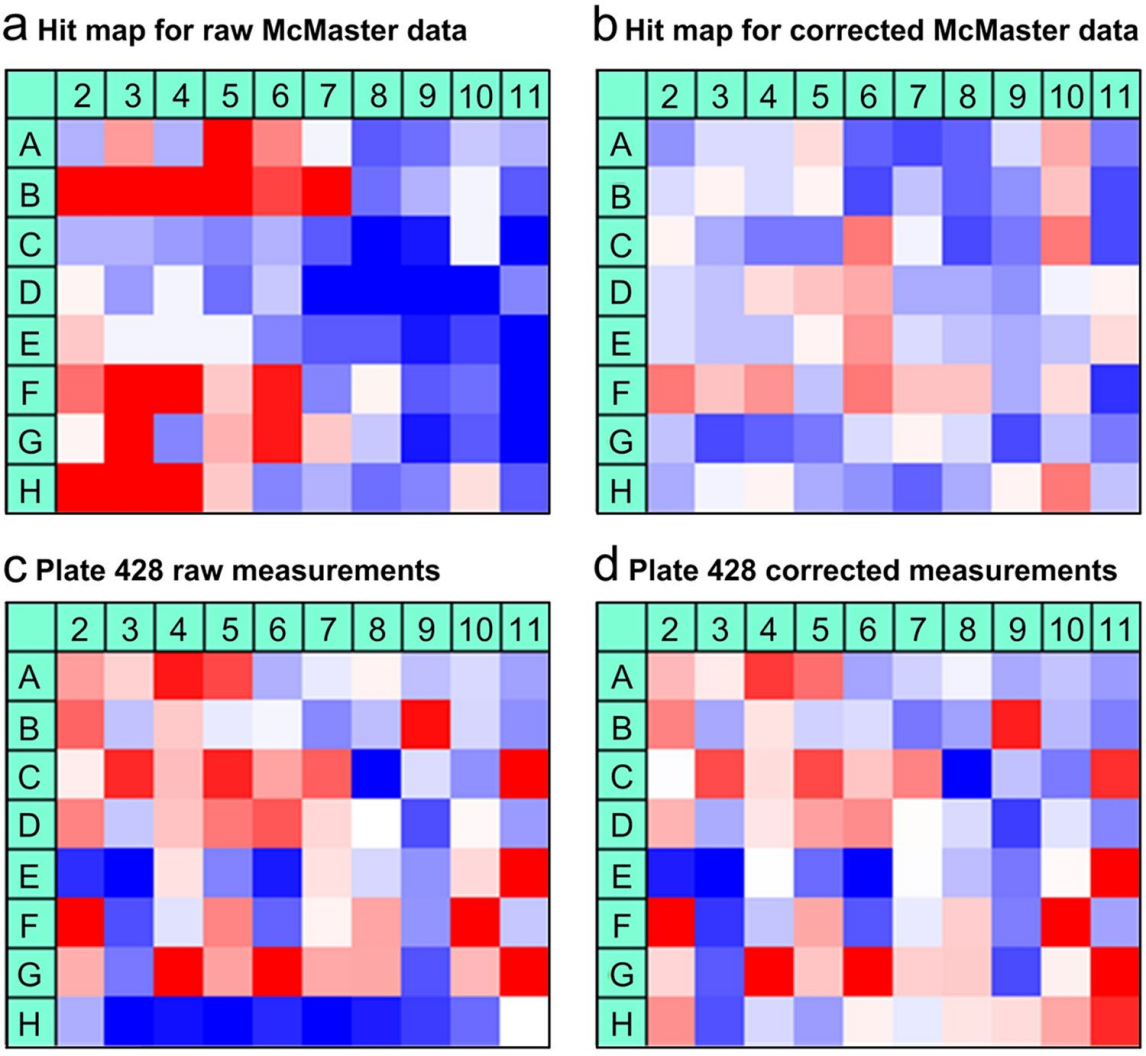
Hit maps showing the presence of spatial bias in the McMaster Test assay screened during the McMaster Data Mining and Docking Competition^13^: (**a**) hit distribution surface for raw data, (**b**) hit distribution surface corrected both plate and assay-wise, (**c**) Plate 428 raw measurements, and (**d**) Plate 428 corrected measurements. Control columns 1 and 12 are not shown here. Higher hit counts (panels a and b) and intensity levels (panels c and d) are in red; lower hit counts (panels a and b) and intensity levels (panels c and d) are in blue. The hit selection threshold of *µ*-2*σ* was used to compute the hit distribution surfaces. The Mann-Whitney *U* test carried out to detect plate-specific spatial bias suggested that 377 McMaster plates were affected by systematic error, and 873 of them were clean. Error detection was done at the significance level *α* = 0.01. Plate-specific spatial bias was corrected using the additive PMP algorithm, as suggested by our method. The exact values of the raw and corrected measurements of Plate 428 and the raw and the corrected hit distribution surfaces are reported in Supplementary Tables 3–6.

Hit distribution surfaces, representing hit counts per well location, were obtained for raw (Fig. 5a) and corrected (Fig. 5b) McMaster measurements using the HTS Corrector software^11^. The positional assay-wide pattern can be observed in raw data as the left-sided wells (they are in red) of the raw hit distribution surface overestimate the number of hits, while the right-sided wells (they are in blue) underestimate it. Additive plate-specific bias correction, followed assay-specific well correction, allowed us to remove spatial bias from raw McMaster data and disrupt the original hit count patterns (Fig. 5b). The *χ*^2^ goodness-of-fit test carried out for the raw McMaster hit distribution surface (Fig. 5a) at the significance level *α* = 0.01 returned the value of *χ*^2^(79) = 416.23 (with the critical value of 111.14), whereas the value of the *χ*^2^ statistic for the corrected hit distribution surface (Fig. 5b), computed following both plate-specific and assay-specific bias corrections, was equal to *χ*^2^(79) = 70.81, showing a much more homogeneous hit distribution pattern (where 79 was the number of degrees of freedom). The *χ*^2^ goodness-of-fit test can be used in screening technologies to assess the deviation of the hit distribution surface from the expected (i.e., plane) surface^11^.

Moreover, plate-specific spatial bias can also be observed within certain plates of the McMaster Test assay. For example, a clear underestimation of the true experimental measurements has occurred in row *H* of Plate 428 of this assay (Fig. 5c). The application of our algorithm presented in the Methods section allowed us to determine that the additive bias model is significant in this case and that it fits the data better than the multiplicative bias model. Using the additive PMP algorithm, we were able to remove systematic error from row *H* without changing the rest of the plate’s measurements (Fig. 5d).

**Figure 6:**
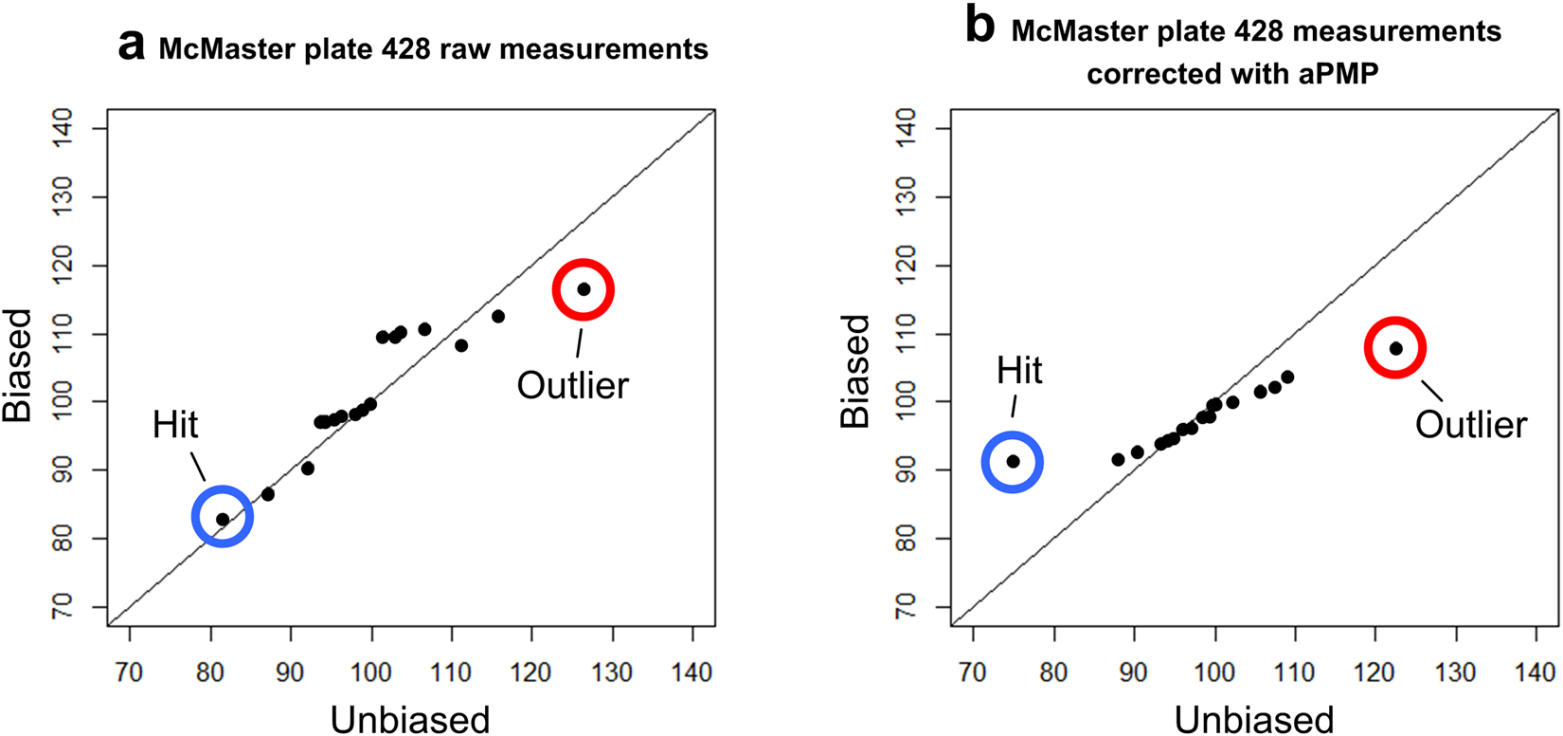
Q-Q plots for McMaster’s Plate 428 from the McMaster Data Mining and Docking Competition HTS Test assay^13^ before (**a**) and after (**b**) the correction of additive spatial bias. All control wells (columns 1 and 12 of each plate) were excluded from the analysis. Low values (i.e., false positives) appearing in row *H* prevent a clear-cut identification of the hit located in well (*E*,3) (panel a of the figure; see also Supplementary Table 5 for the exact values of the raw HTS measurements). After the plate-specific correction by additive PMP, the hit appearing in well (*E*,3) becomes much better separated from the rest of the measurements of Plate 428 (panel b of the figure; see also Supplementary Table 6 for the exact values of the corrected HTS measurements). The same trend is maintained for the outlier (i.e., a high measurement value in the McMaster inhibition assay) located in well (*E*,11).

Moreover, the Q-Q plots in Fig. 6a and b show that the spatial bias correction leads to a much better identification of hits and outliers. Figure 6a presents the Q-Q plot for the raw measurements of Plate 428 of the McMaster Test assay. The hit, located in well (*E*,3) and highlighted by a blue circle in Fig. 6a, and the outlier, located in well (*E*,11) and highlighted by a red circle in this figure, are not well separated from the rest of the data (see also Supplementary Table 5 reporting the raw measurement values of this plate). Thus, both the hit and the outlier cannot be clearly identified using conventional hit selection methods. After the application of the Mann-Whitney *U* test to raw measurements of Plate 428, row *H* was flagged as biased and then corrected plate-wise using the additive PMP algorithm. Following the plate-specific correction by additive PMP, both the hit and the outlier became much better separated from the rest of the data of this plate (see Fig. 6b and Supplementary Table 6 reporting the corrected measurement values of Plate 428) and can be now better identified using conventional hit selection methods.

## Discussion

Over the past decade, a number of novel screening technologies for processing large-scale binding assays have been developed to address the growing needs of drug discovery campaigns. Spatial bias remains one of the major hurdles of most of these technologies, potentially resulting in the identification of false positive and/or false negative hits. Our main objective was to assess the prevalence of spatial bias in experimental data generated by different high-throughput screening technologies.

First, we should distinguish between assay-specific and plate-specific types of spatial bias. Assay-specific bias affects all the plates, or a series of continuous plates (i.e., batch effect), of a given assay. Plate-specific bias manifests itself by a spatial pattern that appears within a given plate only. Using experimental data from the largest public small-molecule screens database, ChemBank^15^, involving three popular screening technologies – high-throughput screening, high-content screening and small-molecule microarrays, and eight of their categories, we found that both assay and plate-specific biases affect the data of most of high-throughput assays. We showed that both assay-specific and plate-specific biases can be recognized and removed successfully from experimental screening data using appropriate statistical methods.

Second, researchers should pay particular attention to the nature of spatial bias that can be additive, multiplicative or undetermined (i.e., when the presence of bias was detected by a statistical test, but both additive and multiplicative bias models were not significant). The presence of assay-specific bias, either additive or multiplicative, in a given well location can be detected by using the nonparametric Mann-Whitney U test. It is worth noting that Z-score normalization adequately handles both additive and multiplicative types of assay-specific bias when applied to the measurements of a given well location (see the Methods section).

However, the case of plate-specific bias is more complicated. The Mann-Whitney U test can be used first to detect the presence of spatial bias in rows and/or columns of a given plate. Then, we can determine whether the detected bias is additive or multiplicative, using the additive and multiplicative versions of the Partial Median Polish (PMP) algorithm (see the Methods section). Afterwards, the Kolmogorov-Smirnov two-sample test should be applied to compare the distributions of unbiased and corrected (by additive and multiplicative PMP) measurements and the *p*-values should be computed for both bias models. The selection of the best-fitting model can be done by comparing the obtained *p*-values.

Considering the example of the McMaster Test assay, we showed that both assay and plate-specific types of spatial bias can be detected and eliminated from screening data, thus improving measurement accuracy and minimizing the risk of misidentifying hits and outliers. It is important to note that the presented methodology is of a generic nature and can be used by life scientists involved in the analysis of current or next-generation high-throughput assays consisting of a large series of plates processed in sequence.

Our simulation study (see the section Simulation study) indicate that the B-score^7^ method is not suitable for removing assay-specific spatial bias and that Well Correction^11^ cannot be used to eliminate plate-specific spatial bias. Thus, these methods should be used only when the presence of bias in the data has been confirmed by an appropriate statistical test (e.g., Mann-Whitney *U* test). The main advantage of our method is that it can cope with both of these biases (i.e., assay and plate-specific). However, the proposed method still needs to be used with caution. Before using it for data correction, the researcher must make sure that all tested compounds are randomly distributed both across and within HTS plates. If the randomization condition is not satisfied, some well locations or some areas on the plates can correctly lower or higher signals, in which case data normalization can become detrimental.

Alternative approaches could also be used to remove spatial bias from data produced by high-throughput screening technologies. For example, one can consider generalized spatial linear mixed models (GLMMs), which are commonly used in public health, ecological and epidemiological studies dealing with geographical sampling^23,24^. GLMMs explicitly model space-dependent bias through a specific spatial correlation function. In the linear mixed model, all observations are assumed to have a spatial correlation structure. A GLMM effectively postulates that the correlation increases as observations are located closer together, whereas observations at distances greater than the range from one another are uncorrelated. The major reason that GLMMs have not been used for bias modeling in HTS is that only a few columns and rows of a given HTS plate are generally biased, whereas mixed models assume that spatial correlation can be modeled in some way throughout the whole plate surface. Moreover, the biased columns and rows are not necessarily spatially related and may not be located at the same part of a given plate. For example, a column or row effect often results from a robot malfunction that is unique to that column or row^25^. Furthermore, in order to accurately model the bias structure in HTS assays, spatial covariates should take into account the dependences between biased rows and columns (i.e., plate-specific bias) as well as between biased well locations (i.e., assay-specific bias) of a given assay. To the best of our knowledge, this type of GLMM still needs to be developed, and this could be an interesting topic for future investigation. It is also known that fitting a GLMM to a large dataset can be computationally expensive (i.e., due to inversion of large matrices growing with the number of sampling units). In contrast, the time complexity of the additive and multiplicative PMP algorithms is linear on the matrix dimensions (see the Methods section). These algorithms require O(*mnI*) time, where *m* and *n* are the plate dimensions and *I* is the number of iterations necessary for convergence. In practice, these algorithms converge after a few iterations.

## Methods

### Correction of assay-specific bias

Assay-specific bias correction methods rely on both plate-wise and well-wise normalization techniques^11^.

First, each plate of the given assay is subject to the robust (outlier-resistant) *Z*-score normalization:

1 $${\hat{x}}_{ijp}=\frac{{x}_{ijp}-me{d}_{p}}{MA{D}_{p}},$$

where *x*_*ijp*_ is the raw measurement in well (*i,j*) of plate *p*, ${\hat{x}}_{ijp}$ is the normalized value of *x*_*ijp*_, and *med*_*p*_ and *MAD*_*p*_ are the median and the median absolute deviation of plate *p*’s measurements, respectively.

Second, the Mann-Whitney *U* test can be carried out for each well location, comparing its sum of ranks to the sum of ranks of the rest of the assay measurements.

Third, the well locations in which the presence of spatial bias was detected by the Mann-Whitney *U* test (the significance level *α* = 0.01 was used in this work) can be corrected via the traditional *Z*-score normalization, defined as follows:

2 $${\hat{x}}_{ijp}=\frac{{x}_{ijp}-{\mu }_{ij}}{{\sigma }_{ij}},$$

where *x*_*ijp*_ is the raw measurement in well (*i,j*) of plate *p*, ${\hat{x}}_{ijp}$ is the normalized value of *x*_*ijp*_, and *μ*_*ij*_ and *σ*_*ij*_ are, respectively, the mean and the standard deviation of the measurements of well location (*i,j*) (computed over all plates of the assay).

Note that the traditional *Z*-score normalization (2) adequately handles both additive and multiplicative kind of biases when applied to the measurements of a given well location. Additive biases are removed by the subtraction in the numerator; the standard deviation does not change if the mean is shifted. In case of multiplicative biases, both the numerator and denominator are multiplied by the same value, and thus the computed score remains the same. Hence, *Z*-score can successfully correct both additive and multiplicative types of assay-specific spatial bias over the measurements of a given well location. Importantly, if the presence of assay-specific bias was detected in some well locations of the assay, then all of its well locations should be *Z*-score normalized in order to keep the assay data on the same scale.

### Correction of plate-specific bias

Plate-specific biases that affect rows and columns of a given plate *p* can fit either the additive (3) or the multiplicative (4) bias model:

3 $$x{^{\prime} }_{ijp}={x}_{ijp}+{r}_{ip}+{c}_{jp}+{\varepsilon }_{ijp},$$

4 $${x^{\prime} }_{ijp}={x}_{ijp}\times {r}_{ip}\times {c}_{jp}+{\varepsilon }_{ijp},$$

where $x{^{\prime} }_{ijp}$ is the observed raw measurement in well (*i,j*) of plate *p*, *x*_*ijp*_ is the real measurement value in well (*i,j*) of plate *p*, *r*_*ip*_ is the value of systematic error (i.e., spatial bias) affecting row *i* of plate *p*, *c*_*jp*_ is the value of systematic error affecting column *j* of plate *p*, and *ε*_*ijp*_ is the random error affecting well (*i,j*) of plate *p*. Partial mean polish, which is a variation of Tukey’s median polish, iteratively removes spatial bias from biased rows and columns^8^. In our work, the presence of bias in rows and columns of each plate *p* was determined using the Mann-Whitney *U* test (with *α* = 0.01). The biased and unbiased rows and columns are identified during the bias detection procedure. The process starts by assuming that all measurements of a given plate are unbiased. The method then compares in turn the measurements of each row and each column against the rest of the plate’s measurements, excluding those of the tested row or column and those that have been already identified as biased using the Mann-Whitney *U* test. The obtained *p*-value is retained for each row and each column; the plate’s row or column corresponding to the smallest *p*-value is then considered biased if this smallest *p*-value is larger than the selected threshold. The procedure is applied independently to all plates of a given assay. A similar procedure is carried out to identify biased well locations (see above).

### Adequate model selection for plate-specific bias

The goodness-of-fit of the additive and multiplicative bias models can be assessed by comparison of the corrected results with unbiased data. For each plate, the process starts with three data sets: (1) measurements from unbiased rows and columns, (2) measurements corrected using the additive correction, and (3) measurements corrected using the multiplicative correction. Afterwards, we carry out the Kolmogorov-Smirnov two-sample test in order to compare the distributions of unbiased measurements (1) and those corrected using the additive correction model (2), then the distributions of unbiased measurements (1) and those corrected using the multiplicative correction model (3). The obtained *p*-values are then compared to each other to select the best fitting model. The significance level *α* = 0.01 was used in our study. The exact algorithm can be presented as follows:

Perform the Mann–Whitney *U* test on each individual plate of the assay (i.e., plate-wise correction) to identify biased rows and columns:

If (spatial bias is detected in rows or columns of the given plate), then:

- Apply the additive PMP algorithm^8^ (see also the next section) to correct raw plate’s measurements;
- Apply the multiplicative PMP algorithm^26^ (see also the next section) to correct raw plate’s measurements;
- Apply the Kolmogorov-Smirnov two-sample test on the corrected plates yielded by the additive and multiplicative PMP algorithms. Compute the corresponding *p*-values;
- If both additive and multiplicative *p-*values are lower than the selected significance level *α*, then the bias model for this plate is undetermined. Otherwise, apply the correction algorithm that provides the largest *p*-value (i.e., additive or multiplicative PMP) to remove spatial bias from the measurements of the given plate.

### Additive and multiplicative Partial Mean Polish (PMP) algorithms

The additive version of the PMP algorithm is presented in detail in the paper of Dragiev and colleagues^8^. Here we show the changes that should be made to the algorithm to adapt it for removing multiplicative biases^26^. The main advantages of both additive^8^ and multiplicative^26^ PMP algorithms are that they correct the biased measurements only and keep the original and corrected data on the same scale, ensuring that the mean of the corrected rows and columns is equal to the mean of unbiased data.

1. Let $R=\{{r}_{1},{r}_{2},\mathrm{...},{r}_{p}|0\le p\le m\}$ and $C=\{{c}_{1},{c}_{2},\mathrm{...},{c}_{s}|0\le s\le n\}$ be the sets of biased rows and columns of plate $P(m\times n)$, calculate the mean *µ* of all unbiased measurements of *P*:
  5 $$\mu =\frac{\sum _{i\notin R,j\notin C}{x}_{ij}}{(m-p)(n-s)}.$$
2. For each biased row *i* in $I=\{1\le i\le p\}$, compute the mean value, ${\mu }_{{r}_{i}}$, of row *r*_*i*_: ${\mu }_{{r}_{i}}=\frac{1}{n}\sum _{j=1}^{n}{x}_{{r}_{i}j}$, and calculate the estimate of the row error, ${\hat{e}}_{{r}_{i}}$, using the equations: ${\hat{e}}_{{r}_{i}}={\mu }_{{r}_{i}}-\mu$(additive model) and ${\hat{e}}_{{r}_{i}}=\frac{|{\mu }_{{r}_{i}}|}{\mu }$(multiplicative model).
  For each biased column *j* in $J=\{1\le j\le s\}$, compute the mean value, ${\mu }_{{c}_{j}}$, of column *c*_*j*_: ${\mu }_{{c}_{j}}=\frac{1}{m}\sum _{i=1}^{m}{x}_{i{c}_{j}}$, and calculate the estimate of the column error, ${\hat{e}}_{{c}_{j}}$, using the equations: ${\hat{e}}_{{c}_{j}}={\mu }_{{c}_{j}}-\mu$ (additive model) and ${\hat{e}}_{{c}_{j}}=\frac{|{\mu }_{{c}_{j}}|}{\mu }$ (multiplicative model).
3. For all rows affected by spatial bias, adjust their measurements using the error estimates determined in Step 2, i.e., for all *i* in $I=\{1\le i\le p\}$ and all *j* such that $1\le j\le n$ proceed as follows: ${x}_{{r}_{i}j}={x}_{{r}_{i}j}-{\hat{e}}_{{r}_{i}}$(additive model) and ${x}_{{r}_{i}j}=\frac{{x}_{{r}_{i}j}}{{\hat{e}}_{{r}_{i}}}$(multiplicative model).
  For all columns affected by spatial bias, adjust their measurements using the error estimates determined in Step 2, i.e., for all *j* in $J=\{1\le j\le s\}$ and all *i* such that $1\le i\le m$ proceed as follows: ${x}_{i{c}_{j}}={x}_{i{c}_{j}}-{\hat{e}}_{{c}_{j}}$(additive model) and ${x}_{i{c}_{j}}=\frac{{x}_{i{c}_{j}}}{{\hat{e}}_{{c}_{j}}}$ (multiplicative model).
4. If $\sum _{i=1}^{p}|\,\,{\hat{e}}_{{r}_{i}}\,|+\sum _{j=1}^{s}|\,{\hat{e}}_{{c}_{j}}| > \varepsilon$, then go to Step 2, otherwise stop the algorithm.

Here *ε* is a small fixed positive threshold.

It is worth noting that the median can be used instead of the mean in both additive and multiplicative PMP algorithms. The use of the median usually increases the method’s robustness against outliers.

### Data availability

High-throughput screening assays analyzed in this study are available in the ChemBank repository^15^. The ChemBank IDs of these assays are indicated in Supplementary Information. The McMaster Data Mining and Docking Competition assay data are available at: http://www.info2.uqam.ca/~makarenkov_v/HTS/home.php.

## Electronic supplementary material

SUPPLEMENTARY INFORMATION
