# Supplementary material for: Identification and correction of spatial bias are essential for obtaining quality data in high-throughput screening technologies
Source: Sci Rep. 2017 Sep 20;7:11921. doi: 10.1038/s41598-017-11940-4 (PMC5607347; doi:10.1038/s41598-017-11940-4)
Supplement: Supplementary file 1 — SUPPLEMENTARY INFORMATION [file 41598_2017_11940_MOESM1_ESM.pdf]

Supplementary Information

Title: Identification and correction of spatial bias are essential for obtaining quality data in high-throughput screening technologies

Bogdan Mazoure[1], Robert Nadon[1,2] & Vladimir Makarenkov[4]*

[1]Department of Computer Science, McGill University, Montréal, Canada.

[2]Department of Human Genetics, McGill University, Montréal, Canada.

[3]McGill University and Genome Quebec Innovation Centre, Montréal, Canada.

[4]Department of Computer Science, Université du Québec à Montréal, Montréal, Canada.

*Correspondence should be addressed to V.M. (makarenkov.vladimir@uqam.ca).

Supplementary Information

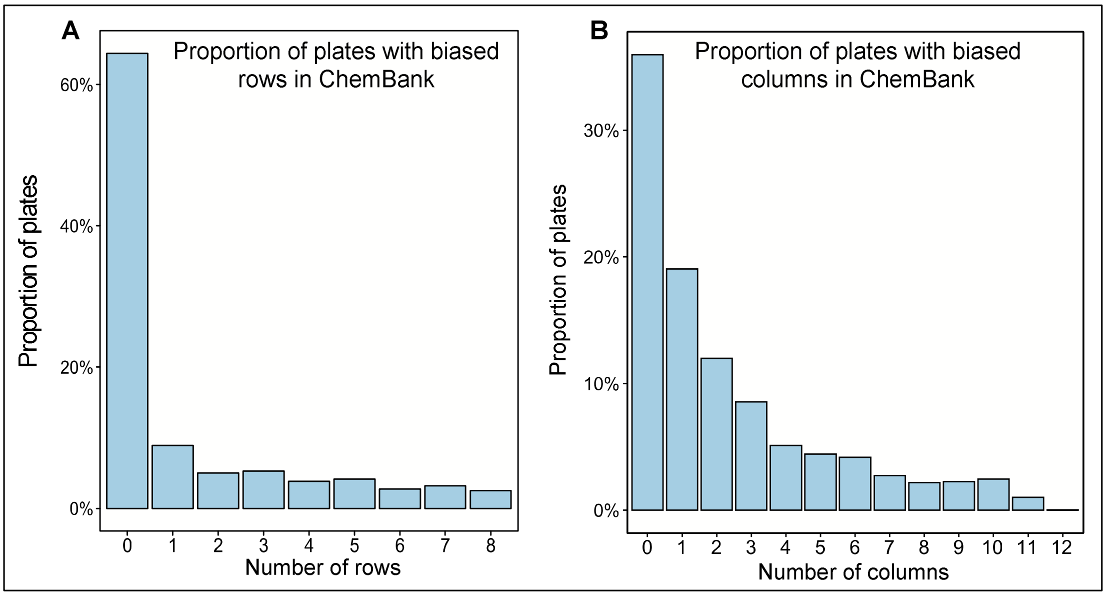

Supplementary Figure 1. Distributions of the number of rows (A) and columns (B) per plate affected by spatial bias, computed over the 2441 non-empty plates of the 175 ChemBank assays analyzed in this study (see Supplementary Table 2). The non-parametric Mann-Whitney *U* test with the cut-off level $\alpha = 0.01$ was used to detect the presence of spatial bias within each plate.

Supplementary Table 1: Set of 12 ChemBank assays examined in our assay-specific bias detection simulation (see **Fig. 2**).

	**Assay name**	**ChemBank ID**	**Number of plates**
**High-throughput screening– 4 assays**			
**1**	ABeta42 aggregation inhibitors	1103.0016	30
**2**	Bacterial viability profiling	1064.0002	10
**3**	*E. coli* filamentation	1038.0004	17
**4**	*M. tuberculosis* sulfur assimilation	130.0018	51
**High-content screening – 4 assays**			
**1**	Autophagy cell count	1050.0009	7
**2**	Autophagy EGFP	1050.0111	8
**3**	Toxoplasma invasion imaging screening	141.0027	38
**4**	*C. elegans* assay for anti-infective reagents	1109.0003	5
**Small-molecule microarrays – 4 assays**			
**1**	HPV-E7 SMM	1049.0001	27
**2**	Male germ cell targets SMM	1154.0015	27
**3**	Male germ cell targets SMM	1154.0009	27
**4**	NeuroSMM screen on torsin A	1069.0001	27

Supplementary Table 2: Set of 175 ChemBank assays examined in our plate-specific bias detection simulation (see **Figs. 3** and **4**). Note that only 8 non-empty HCS Area, 18 non-empty HCS Intensity and 24 non-empty HCS Cell count assays were available in ChemBank (as of November 30th, 2016). For all other screening categories, 25 assays per data category were examined.

**High-throughput screening (Cell-based) – 25 assays**	
**1**	AdipocyteDifferentiation1_OilRedO(913.0191)
**2**	AdipocyteDifferentiation2_NileRed(1015.0001)
**3**	AdipocyteDifferentiation2_NileRed(1015.0032)
**4**	AdipocyteDifferentiation2_NileRed(1015.0034)
**5**	AnnotationDevelopment_BrdUCytoblot(900.0001)
**6**	AnnotationDevelopment_BrdUCytoblot(900.0002)
**7**	AnnotationDevelopment_BrdUCytoblot(900.0021)
**8**	AnnotationDevelopment_BrdUCytoblot(900.0022)
**9**	AnnotationDevelopment_EthD1Staining(900.0005)
**10**	AnnotationDevelopment_JC1MitoDye(900.0013)
**11**	AR-NcoRBindingAssay_raw{Lux()}(268.0159)
**12**	AR-NcoRBindingAssay_raw{Lux()}(268.0173)

**13**	AR-NcoRBindingAssay_raw{Lux()}(268.0217)
**14**	AR-NcoRBindingAssay_user{AvgLux()}(268.0221)
**15**	BreastCancerCellProfiling_CellTiterGlo(915.0248)
**16**	BreastCancerCellProfiling_JC1MitoDye(915.0244)
**17**	CellularAutofluorescence_CpdAutofluor(908.0049)
**18**	CellularAutofluorescence_CpdAutofluor(908.0125)
**19**	CellViabilityProfiling_CellTiterGlo(1019.0001)
**20**	DeacetylaseInhibition_AcLysCytoblot(1027.0002)
**21**	EndothelialCellProfiling1_Calcein-AM(910.0153)
**22**	FacioscapulohumeralMD_Calc(E1-E2)(1026.0010)
**23**	FacioscapulohumeralMD_Calc(E1-E2)(1026.0011)
**24**	FacioscapulohumeralMD_LuxReporter(1026.0003)
**25**	FacioscapulohumeralMD_LuxReporter(1026.0019)
**High-throughput screening (Homogeneous) – 25 assays**	
**1**	ActinPolymerization_raw{FI()}(144.0030)
**2**	ActinPolymerization_user{Fold()}(144.0031)
**3**	AdipocyteDifferentiation1_OilRedO(913.0190)
**4**	AnnotationDevelopment_BrdUCytoblot(900.0020)
**5**	BRAF_HRPCytoblot(1110.0001)
**6**	BRAF_HRPCytoblot(1110.0002)
**7**	BRAF_HRPCytoblot(1110.0003)
**8**	BRAF_HRPCytoblot(1110.0005)
**9**	CellularAutofluorescence_CpdAutofluor(908.0050)
**10**	CMVPolymeraseBindingAssay_raw{Pol(P)}(299.0552)
**11**	CMVPolymeraseBindingAssay_raw{Pol(s)}(299.0543)
**12**	CMVPolymeraseBindingAssay_raw{Pol(s)}(299.0549)
**13**	CMVPolymeraseBindingAssay_raw{Pol(s)}(299.0553)
**14**	CREBReporterAssay_LacZReporter(1029.0010)
**15**	CyclinReporterGeneCdh1_user{Fold()}(219.0091)
**16**	DihydroorotateDehydrogenase_Calc(E1-E2)(1021.0033)
**17**	DihydroorotateDehydrogenase_EnzCoupledColor(1021.0001)
**18**	DihydroorotateDehydrogenase_EnzCoupledColor(1021.0013)
**19**	EColiFilamentation2006_OpticalDensity(1038.0010)
**20**	GlycanaseActivity_raw{Pol(P)}(295.0495)
**21**	HoxDNA-BindingAssay_FluorOligo(1031.0002)
**22**	HoxDNA-BindingAssay_FluorOligo(1031.0008)
**23**	HoxDNA-BindingAssay_FluorOligo(1031.0010)
**24**	KinaseInhibitorModifiers_BrdUCytoblot(901.0010)
**25**	TrypanothioneReductase_EnzCoupledColor(1017.0020)
**High-throughput screening (Microorganism) – 25 assays**	
**1**	ABAggregationInhibitors_OpticalDensity(1103.0009)
**2**	AntibacterialAssay_FluorProtein(1106.0016)
**3**	AntibacterialAssay_FluorProtein(1106.0027)
**4**	AspulvinoneUpregulation_MetabColor(1022.0007)
**5**	BiofilmFormationAssay_BacTiterGlo(1059.0006)
**6**	ClathrinDependentMembraneTrafficking_raw{Abs(Mut)}(310.0609)
**7**	ClathrinDependentMembraneTrafficking_raw{Abs(Mut)}(310.0613)
**8**	ClathrinDependentMembraneTrafficking_raw{Abs(Mut)}(310.0625)
**9**	ClathrinDependentMembraneTrafficking_raw{Abs(Mut)}(310.0657)
**10**	ClathrinDependentMembraneTrafficking_raw{Abs(Mut)}(310.0737)
**11**	ClathrinDependentMembraneTrafficking_user{Fold(Mut)}(310.0733)
**12**	EColiFilamentation2006_OpticalDensity(1038.0001)
**13**	EColiFilamentation2006_OpticalDensity(1038.0002)
**14**	EColiFilamentation2006_OpticalDensity(1038.0012)
**15**	EColiFilamentation2006_OpticalDensity(1038.0014)
**16**	EColiFilamentation2006_OpticalDensity(1038.0016)
**17**	EColiFilamentation2006_OpticalDensity(1038.0022)
**18**	EColiFilamentation2006_OpticalDensity(1038.0023)
**19**	EColiFilamentation2006_OpticalDensity(1038.0024)
**20**	PDERegulators_OpticalDensity(1091.0043)
**21**	PhosphatidylinositolKinase_OpticalDensity(1000.0008)
**22**	PhosphatidylinositolKinase_OpticalDensity(1000.0027)
**23**	PSACAntagonistScreen_OpticalDensity(1035.0016)
**24**	SulfurAssimilation_user{Inh(BioB)}(130.0018)
**25**	SulfurAssimilation_user{Inh(CysH)}(130.0020)
**High-throughput screening (Gene expression) – 25 assays**	
**1**	AndrogenSignalingGE-HTS_GeneExprHTS(1004.0001)
**2**	AndrogenSignalingGE-HTS_GeneExprHTS(1004.0003)
**3**	AndrogenSignalingGE-HTS_GeneExprHTS(1004.0007)
**4**	AndrogenSignalingGE-HTS_GeneExprHTS(1004.0009)
**5**	AndrogenSignalingGE-HTS_GeneExprHTS(1004.0010)
**6**	AndrogenSignalingGE-HTS_GeneExprHTS(1004.0012)

**7**	AndrogenSignalingGE-HTS_GeneExprHTS(1004.0014)
**8**	AndrogenSignalingGE-HTS_GeneExprHTS(1004.0016)
**9**	AndrogenSignalingGE-HTS_GeneExprHTS(1004.0017)
**10**	AndrogenSignalingGE-HTS_GeneExprHTS(1004.0019)
**11**	AndrogenSignalingGE-HTS_GeneExprHTS(1004.0021)
**12**	AndrogenSignalingGE-HTS_GeneExprHTS(1004.0028)
**13**	AndrogenSignalingGE-HTS_GeneExprHTS(1004.0030)
**14**	GE-HTSApoptosis_GeneExprHTS(1055.0005)
**15**	GE-HTSApoptosis_GeneExprHTS(1055.0015)
**16**	GE-HTSNotch1Inhibition_GeneExprHTS(1131.0083)
**17**	GE-HTSNotch1Inhibition_GeneExprHTS(1131.0089)
**18**	GE-HTSNotch1Inhibition_GeneExprHTS(1131.0092)
**19**	MetabolismCellProfiling_GeneExprHTS(1020.0071)
**20**	MetabolismCellProfiling_GeneExprHTS(1020.0078)
**21**	NeuroblastomaDifferentiation_GeneExprHTS(1149.0007)
**22**	NeuroblastomaDifferentiation_GeneExprHTS(1149.0016)
**23**	NeuroblastomaDifferentiation_GeneExprHTS(1149.0039)
**24**	NeuroblastomaDifferentiation_GeneExprHTS(1149.0046)
**25**	NeuroblastomaDifferentiation_GeneExprHTS(1149.0047)
**High-content screening (Area) – 8 assays**	
**1**	Autophagy_AvVesicleArea(1050.0019)
**2**	Autophagy_AvVesicleArea(1050.0064)
**3**	Autophagy_AvVesicleArea(1050.0077)
**4**	Autophagy_AvVesicleArea(1050.0116)
**5**	Autophagy_VesicleAreaPerCell(1050.0016)
**6**	Autophagy_VesicleAreaPerCell(1050.0079)
**7**	Autophagy_VesicleTotalArea(1050.0015)
**8**	Autophagy_VesicleTotalArea(1050.0084)
**High-content screening (Intensity) – 18 assays**	
**1**	Autophagy_EGFPVes7(1050.0076)
**2**	Autophagy_VesicleAvInt(1050.0018)
**3**	Autophagy_VesicleAvInt(1050.0067)
**4**	Autophagy_VesicleAvInt(1050.0080)
**5**	Autophagy_VesicleInt(1050.0017)
**6**	Autophagy_VesicleInt(1050.0083)
**7**	BetaCatenin_AvgIn(1152.0003)
**8**	BetaCatenin_AvgIn(1152.0007)
**9**	BetaCatenin_PosCellsW2_Int(1152.0015)
**10**	BetaCatenin_PosCellsW2_Int(1152.0019)
**11**	BetaCatenin_W2AvgIntPosNuc(1152.0002)
**12**	DNADamageImagingScreen_pChk1AvNucInt(1037.0008)
**13**	DNADamageImagingScreen_pChk1AvNucInt(1037.0010)
**14**	KLF2Regulators_KLF2-GFP_AvInt(1085.0006)
**15**	KLF2Regulators_KLF2-GFP_Int(1085.0005)
**16**	ProteinDegradationInhibition_MODCInt(1053.0006)
**17**	ProteinDegradationInhibition_MODCInt(1053.0036)
**18**	ProteinDegradationInhibition_MODCInt(1053.0044)
**High-content screening (Cell count) – 24 assays**	
**1**	Autophagy_CellCount(1050.0007)
**2**	Autophagy_CellCount(1050.0065)
**3**	Autophagy_CellCount(1050.0078)
**4**	Autophagy_CellCount(1050.0117)
**5**	Autophagy_CellCountSupra10Punctae(1050.0010)
**6**	Autophagy_CellCountSupra15Punctae(1050.0011)
**7**	Autophagy_CellCountSupra20Punctae(1050.0012)
**8**	Autophagy_CellCountSupra5Punctae(1050.0008)
**9**	Autophagy_CellCountSupra7Punctae(1050.0009)
**10**	Autophagy_VesicleCount(1050.0014)
**11**	Autophagy_VesicleCount(1050.0081)
**12**	Autophagy_VesicleCountPerCell(1050.0013)
**13**	Autophagy_VesicleCountPerCell(1050.0082)
**14**	KLF2Regulators_CellCount(1085.0003)
**15**	StemCellChemicalBiology_CellCount(1032.0652)
**16**	StemCellChemicalBiology_CellCount(1032.0657)
**17**	StemCellChemicalBiology_CellCount(1032.0660)
**18**	StemCellChemicalBiology_CellCount(1032.0663)
**19**	StemCellChemicalBiology_CellCount(1032.0666)
**20**	StemCellChemicalBiology_LiveCells(1032.0653)
**21**	StemCellChemicalBiology_LiveCellsPerCellCount(1032.0654)
**22**	StemCellChemicalBiology_Sox17PosPerCellCount(1032.0659)
**23**	StemCellChemicalBiology_Sox17PosPerCellCount(1032.0662)
**24**	StemCellChemicalBiology_Sox17PosPerCellCount(1032.0668)

Small-molecule microarrays – 25 assays	
**1**	Abeta40SMM_AutoSNR(1115.0001)
**2**	Abeta40SMM_AutoSNR(1115.0002)
**3**	Abeta40SMM_AutoSNR(1115.0003)
**4**	Abeta40SMM_AutoSNR(1115.0004)
**5**	CFTRSMM_ManualSNR(1098.0001)
**6**	CFTRSMM_ManualSNR(1098.0002)
**7**	CFTRSMM_ManualSNR(1098.0004)
**8**	CFTRSMM_ManualSNR(1098.0005)
**9**	CFTRSMM_ManualSNR(1098.0006)
**10**	DHODHSMM_ManualSNR(1089.0001)
**11**	EBNA1SMM_ManualSNR(1159.0001)
**12**	HIV-1NefSMM_ManualSNR(1150.0001)
**13**	HIV-1NefSMM_ManualSNR(1150.0002)
**14**	HPVE7SMM_ManualSNR(1049.0001)
**15**	LRP130_ManualSNR(1140.0001)
**16**	LRP130_ManualSNR(1140.0002)
**17**	LRP130_ManualSNR(1140.0003)
**18**	LRP130_ManualSNR(1140.0007)
**19**	MaleGermCellSMM_ManualSNR(1154.0009)
**20**	MaleGermCellSMM_ManualSNR(1154.0015)
**21**	NeuroSMM_ManualSNR(1069.0001)
**22**	PETLigandSMM_ManualSNR(1153.0001)
**23**	PETLigandSMM_ManualSNR(1153.0012)
**24**	SMMDIV06Annotation_AutoSNR(1066.0013)
**25**	Transcription Factor Profile_AutoSNR(1125.0075)

Supplementary Table 3: McMaster's raw hit distribution surface (**Fig. 5a**). The hit distribution surface is used to report the total number of hits in each well over all plates of the assay. It can be assessed with the χ^2 goodness-of-fit statistic and compared to the expected (i.e., often mean) value (see the main text for more details).

	2	3	4	5	6	7	8	9	10	11
**A**	15	23	15	37	24	18	11	12	16	15
**B**	30	30	42	45	27	41	12	15	18	11
**C**	15	15	14	13	15	11	6	8	18	7
**D**	19	14	18	12	16	7	7	7	7	13
**E**	21	18	18	18	13	11	11	8	10	7
**F**	25	37	36	21	29	13	19	11	12	6
**G**	19	30	13	22	29	21	16	8	11	7
**H**	31	42	42	21	13	15	12	13	20	11

Supplementary Table 4: McMaster's hit distribution surface after the plate and assay-wise data correction (**Fig. 5b**).

	2	3	4	5	6	7	8	9	10	11
**A**	13	16	16	19	11	10	11	16	21	12
**B**	16	18	16	18	10	15	11	13	20	10
**C**	18	14	12	12	23	17	10	12	23	10
**D**	16	15	19	20	21	14	14	13	17	18
**E**	16	15	15	18	22	16	15	14	15	19
**F**	23	20	22	15	23	20	20	14	19	9
**G**	15	10	11	12	16	18	16	10	15	12
**H**	14	17	18	14	13	11	14	18	23	15

Supplementary Table 5: Raw measurements of McMaster's Plate 428 (**Fig. 5c**). The hit value is highlighted in blue and bold, and the three next closest values are highlighted in blue.

	2	3	4	5	6	7	8	9	10	11
**A**	103.84	100.05	108.67	106.56	94.07	96.1	98.47	96.36	97.15	94.6
**B**	104.28	93.37	98.56	94.69	95.22	90.38	94.16	108.06	96.18	94.25
**C**	98.74	106.65	100.49	106.47	101.73	105.24	88.8	96.01	93.28	107.44
**D**	102.16	94.34	99.62	101.81	103.75	99.79	98.03	91.35	97.68	94.69
**E**	87.56	**74.99**	97.15	92.05	87.12	99.79	96.8	93.99	98.74	122.57
**F**	111.14	87.65	93.55	100.23	90.29	96.89	99.88	92.93	107.36	96.01
**G**	99.44	89.5	108.76	99.44	120.28	99.44	98.91	91.61	99.88	117.82
**H**	90.56	77.8	84.13	81.94	86.95	85.98	88.97	89.85	92.67	98.03

Supplementary Table 6: Corrected measurements of McMaster's Plate 428 (**Fig. 5d**). The correction was done using the additive PMP algorithm (the most appropriate in this case). The hit value is highlighted in blue and bold, and the three next closest values are highlighted in blue.

	**2**	**3**	**4**	**5**	**6**	**7**	**8**	**9**	**10**	**11**
**A**	103.84	100.05	108.67	106.56	94.07	96.1	98.47	96.36	97.15	94.6
**B**	104.28	93.37	98.56	94.69	95.22	90.38	94.16	108.06	96.18	94.25
**C**	98.74	106.65	100.49	106.47	101.73	105.24	88.8	96.01	93.28	107.44
**D**	102.16	94.34	99.62	101.81	103.75	99.79	98.03	91.35	97.68	94.69
**E**	87.56	**74.99**	97.15	92.05	87.12	99.79	96.8	93.99	98.74	122.57
**F**	111.14	87.65	93.55	100.23	90.29	96.89	99.88	92.93	107.36	96.01
**G**	99.44	89.5	108.76	99.44	120.28	99.44	98.91	91.61	99.88	117.82
**H**	100.85	88.09	94.42	92.23	97.24	96.27	99.26	100.14	102.96	108.32
